# Perspectives about and approaches to weight gain in pregnancy: a qualitative study of physicians and nurse midwives

**DOI:** 10.1186/1471-2393-13-47

**Published:** 2013-02-21

**Authors:** Tammy Chang, Mikel Llanes, Katherine J Gold, Michael D Fetters

**Affiliations:** 1 Department of Family Medicine, University of Michigan, Ann Arbor, MI, USA; 2 University of Michigan, Robert Wood Johnson Clinical Scholars Program, North Campus Research Complex, 2800 Plymouth Road, Building 10 – Room G016, Ann Arbor, MI, 48109-2800, USA; 3 Department of Family Medicine, Department of Obstetrics & Gynecology, University of Michigan, 1018 Fuller Street, Ann Arbor, MI, 48104-1213, USA; 4 Ypsilanti Health Clinic, University of Michigan, 200 Arnet Street, Suite 200, Ypsilanti, MI, 48198, USA; 5 Department of Family Medicine, University of Michigan, 1018 Fuller Street, Ann Arbor, MI, 48104-1213, USA

**Keywords:** Prenatal care, Gestational weight gain, Obesity, Qualitative study, Obstetrics, Certified nurse midwives, Family physician

## Abstract

**Background:**

Over one third of reproductive age women in the US are obese. Pregnancy is a strong risk factor for obesity, with excess weight gain as the greatest predictor of long term obesity. The majority of pregnant women gain more weight than recommended by the Institute of Medicine guidelines. The objective of this study was to understand prenatal care providers’ perspectives on weight gain during pregnancy.

**Methods:**

Semi-structured qualitative interviews of 10 prenatal care providers (three family physicians, three obstetricians, and four nurse midwives) at a University Hospital in the Midwest, that included the ranking of important prenatal issues, and open-ended questions addressing: 1) general perceptions; 2) approach with patients; and 3) clinical care challenges.

**Results:**

Providers felt that appropriate weight gain during pregnancy was not a high priority. Many providers waited until patients had gained excess weight before addressing the issue, were not familiar with established guidelines, and lacked resources for patients. Providers also believed that their counseling had low impact on patients, avoided counseling due to sensitivity of the topic, and believed that patients were more influenced by other factors, such as their family, habits, and culture.

**Conclusions:**

Both providers and patients may benefit from increased awareness of the morbidity of excess weight gain during pregnancy. Practice-level policies that support the monitoring and management of weight gain during pregnancy could also improve care. Research that further investigates the barriers to appropriate weight gain is warranted.

## Background

Obesity among reproductive age women is a prevalent, debilitating, and expensive public health problem. Obesity is associated with serious physical, psychological and social problems including cardiovascular disease, lower quality of life, and stigma [1-4]. Despite over a decade of focus, overall rates of obesity among reproductive age women in the US remain high, with between a quarter and a third of women age 20–44 categorized as obese [5,6].

Pregnancy is a time of expected weight gain. However, the majority of US women gain more than the recommended weight per Institute of Medicine guidelines [7,8]. In fact, pregnancy itself is a strong risk factor for future obesity, with excess weight gain during pregnancy as the greatest predictor of long term obesity [9-11].

Excess weight gain during pregnancy is associated with serious short and long-term consequences for both mothers and their infants. Risks of excess maternal weight gain to infants include low five- minute APGAR score, seizure, hypoglycemia, hyperbilirubinemia, polycythemia, meconium aspiration syndrome, macrosomia, and childhood overweight [12-14]. Perinatal complications such as miscarriage, Caesarean section, development of diabetes mellitus, pregnancy-induced hypertension, as well postpartum weight retention and overweight are among the adverse consequences of excess weight gain during pregnancy for mothers [10,15,16].

In general, weight management during pregnancy has not been emphasized in the prenatal care of patients. One cross-sectional study of Canadian patients of midwives, family physicians, and obstetricians showed very low rates (5.7%-16.3%) of counseling about gestational weight gain by all types of providers [17]. When information is given antenatally regarding weight gain, the advice is typically brief and generally not related to weight management as reported by a recent study in the UK [18]. Interestingly, despite having the highest rates of excessive weight gain nationally, white women were the least likely to receive counseling about nutrition during pregnancy in a cohort study of predominantly low-income prenatal patients in the US [19].

Studies have examined the patient’s perspective of weight gain during pregnancy. These studies show that gestational weight gain is associated with overall body image [20], and that the desire to return to prepregnancy weight was a strong motivating factor to control weight gain. In addition, the health and well-being of their unborn baby is often central in women’s decisions about appropriate weight gain [21], and women’s attitudes about weight gain in pregnancy are embedded in their overall orientation toward pregnancy and their general psychological functioning [22]. Low-income black women in the US had more perceptions encouraging high gestational weight gain than discouraging it [23]. Furthermore, low-income black women did not limit their gestational weight gain, despite knowledge of the risk for weight retention due to their belief that gaining more weight is indicative of a healthy infant [24].

To better understand the complex problem of excess weight gain during pregnancy, it is vital to understand the perspectives of prenatal care providers as well. The objective of this study was to understand the perceptions, approach, and challenges regarding management of weight gain during pregnancy among a sample of family physicians, obstetricians, and certified nurse midwives who provide prenatal care.

## Methods

### Design

We used a qualitative design employing semi-structured qualitative interviews. This study was approved by the University of Michigan’s institutional review board.

### Setting

Academic medical center in the Midwest with Family Medicine, Nurse Midwifery, and Obstetrics clinical and educational programs [25,26].

### Participants and recruitment

We used maximum variation sampling to obtain a variety of medical specialties and providers with a breadth of background and experience within our sample. Providers were selected based on specialty (FP, OB, CNM) and to represent a broad level of experience (faculty vs resident), and were either contacted by email or face-to-face to participate in our study. From this process, we recruited three family physicians (FP), three obstetricians (OB) and four certified nurse midwives (CNM) who practice at community-based sites as part of a university hospital in the Midwest. All eligible providers that were asked to participate consented to be interviewed for the study (n = 10). No incentive was offered for participation. Verbal informed consent was obtained from all study participants, and documented by audio-recording.

### Data collection

Two investigators (TC, ML) conducted face-to-face, in-depth, semi-structured interviews using an interview guide developed by the authors after the participants provided their verbal informed consent to participate. The interviews began by participants ranking a list of eleven important prenatal issues occurring during a typical prenatal visit to learn their perceptions about the importance of weight gain relative to other common issues. We then asked semi-structured, open-ended questions addressing their general perceptions about weight gain, their clinical approach to weight gain, and challenges they encounter in the management of weight gain during pregnancy. Interviews were performed by two researchers in private rooms and were audio-recorded.

### Standard Interview Guide

Domain 1. Perception of weight gain management

How would you prioritize these prenatal care issues?

1. Smoking

2. Substance abuse

3. Folic acid supplementation

4. Appropriate weight gain

5. Domestic abuse

6. Nutrition/Diet

7. Exercise

8. Stress

9. Mood disturbances

10. STD prevention

11. Round ligament pain

Tell me your thoughts about the management of weight gain during pregnancy?

Probe- Is it a problem? How important is it? Why?

Domain 2. Counseling of patients

What is your general approach to weight gain in pregnancy?

Why do you follow this approach?

Probe – Do you follow any guidelines or general rules?

How do you approach these discussions?

Probe –Timing, specific advice

What resources do you use to help patients?

What factors influence your approach?

Domain 3. Challenges with counseling about weight gain in pregnancy

What is the impact of your weight gain management?

How receptive do you feel your patients are to counseling?

What do you find is most effective?

What do you find is not effective?

What are specific barriers or other challenges?

### Data analysis

Interviews were transcribed verbatim, and reviewed for accuracy. We used inductive qualitative techniques informed by thematic analysis [27]. We began by exploring how providers perceived the management of weight gain during pregnancy and their specific approaches to management. Transcripts were reviewed line by line to identify prominent concepts and ideas to draft preliminary coding categories. These initial findings were reviewed, coding categories were created, and themes were added and clarified as a team. Two researchers (TC, ML) engaged in an inductive process of reading and manually coding two transcripts together. Codes were further clarified and a codebook with definitions was developed. From this codebook, the remaining transcripts were coded independently. Inter-coder agreement was 92%. Team members (TC, ML) reviewed results in frequent meetings and discussions, using memos to identify emerging themes and describe relationships among coding categories [28]. The final coding scheme and analysis of the findings were reviewed, and disagreements were discussed until consensus was reached. We organized the results using the coding scheme structure and illustrated the themes and sub-themes with representative quotations.

To help achieve ‘trustworthiness’ of our results, we performed “member checking” where the overall results of the study were emailed to each participant. Eight of the interviewed providers including at least one provider from each specialty (FP, OB, CNM) responded to the request for member checking. Each of them indicated that the results included and accurately represented their viewpoints.

## Results

### Participant characteristics

Providers included had varying levels of clinical experience and included senior residents and senior faculty members. Most providers reported the composition of their patient population was primarily white. However, several of the providers also worked with populations of patients whose composition was primarily black or diverse (Table 1). All providers were employed by a large academic health system where they provided labor and delivery care, though their outpatient prenatal care was based in local community settings.

**Table 1:** Participant Characteristics, n = 10

**Characteristic**	**n**
**Women**	7
**Obstetricians**	3
**Family Physicians**	3
**Certified Nurse Midwives**	4
Faculty	8
Resident	2
**Race**	
White	9
Black	1
**Racial composition of patients**	
Primarily White	7
Primarily Black	1
Diverse*	2

*Diverse is defined as having nearly equal proportion of white, black and other racial/ethnic groups.

### Overview

The following sections describe major themes around perceptions of the management of weight gain during pregnancy, approaches to management, and challenges faced. Quotations are labeled by participant number. We found no notable differences between specialty groups (FP, CNM, OB) in regards to their perceptions and general approaches to counseling and care.

### Priority of weight gain relative to other common prenatal issues

When asked to verbally rank a list of 11 common prenatal issues (see subsection Standard Interview Guide), some providers ranked the issues numerically, while others discussed only their “top” issues stating that the remainder were less important. Three providers reported that “appropriate weight gain” was not a “top” priority, while assessing smoking, substance abuse, domestic abuse, and mood disturbances as more important.

The highest numerical ranking of “appropriate weight gain” among any of the participants was only 4 out of 11, while five providers ranked it 7 or lower (7 by one provider, 8 by three providers, and 9 by one provider.)

Qualitative comments made by providers from all three specialties further illustrated their belief that weight gain during pregnancy was not a high priority compared to competing issues.

“*…her weight would be last on the priority list.” (Participant 8)*

“I tend not to talk about weight gain.” (1)

### Approaches to care

The approach to weight gain during pregnancy ranged from no approach to a focus on diet and exercise rather than on appropriate weight gain.

“I mention their labs, blood pressure, weight gain as routine. May comment on it, but I may not think it’s an issue. When is it an issue? When do I worry about weight gain? I have to say that I probably don’t focus on the weight gain as a concern as much as nutrition. If they are eating healthy but they are gaining a little more weight than the recommendation, I may mention it to them, but I don’t worry about it.” (6)

When asked about guidelines informing their approach to appropriate weight gain, none stated knowledge of the IOM guidelines, while others described varying ranges of weight gain recommendations they followed that were learned during their training.

“It’s the nutritional guidelines through the governmental regulations for pregnancy- that is the benchmarks.” (4)

“I have a range… if the patient is normal weight, I say a minimum of 15 pounds, maybe 15–25 pounds.”(10)

Although many providers reported that weight gain was not emphasized during routine prenatal care, once excess weight gain was detected, providers would then focus on the issue.

“I guess I might bring it up more if they are gaining a lot of weight.” (9)

Most providers reported a lack of accessible and effective resources for patients, especially nutritional education/counseling. Moreover these providers doubted the efficacy of nutritional counseling or doubted that patients would have access to these resources due to cost and insurance limitations.

“I don’t think dieticians are very successful so I don’t send my patients.” (10)

“(I refer for) nutritional counseling if their insurance will pay for it, but it gets tricky…” (3)

### Attitudes and beliefs

Not only did providers believe that nutritional counseling was ineffective, many providers did not believe their own interventions could change their patients’ behavior.

“No- I don’t think my lame diet counseling makes a difference just the same way that people don’t lose weight even after doctor’s counseling. Is it cultural or habit- yeah.” (7)

“It seems that some doctors really harp on it, but I don’t. I don’t believe we have much control over this.” (7)

Providers perceived sensitivity to discussing weight with their patients and also expressed their own caution in addressing this topic. Many providers wanted to avoid ideas of body image, specifically patients perceiving that their doctor was labeling them.

“It’s probably because it makes me uncomfortable. Probably some of my care is biased because it’s someplace I don’t want to go. I don’t want to tell a patient that they are fat.” (2)

Many providers perceived that patients were more influenced by other factors in regards to weight gain during pregnancy, which was one reason why providers felt they had little impact on a patient’s weight gain.

“The patients that are obese are obese for various reasons and those habits are hard to break. They may have socio-economic factors that make them not be able to afford healthy foods and I think those are not overcomeable very easily.” (9)

“It’s a challenge because it is a cultural issue depending on their family history.” (5)

## Discussion

Our study begins to characterize the complexity of weight gain during pregnancy from the perspective of a variety of prenatal care providers at an academic institution. In addition, we have identified several barriers among prenatal care providers to effective weight management, as well as potential interventions and policy changes to address these challenges (Table 2). Not only does it appear that providers perceive that many women believe that they should be “eating for two” as suggested by past studies [23,24], providers may not consider the management of weight gain during pregnancy as important or effective.

**Table 2:** Barriers to effective weight management during pregnancy among prenatal care providers, and potential interventions and/or policy changes

**Category**	**Theme**	**Representative quote**	**Potential intervention or policy change**
Priority	Low Priority	“I have to say that I probably don’t focus on the weight gain as a concern.” (6)	Increase focus in medical education on morbidity of excess weight gain during pregnancy. Increase awareness through popular media and empower patients to voice their concerns to providers.
Approach to Care	Unfamiliarity with established guidelines	I don’t know if it is right, but in my own mind it’s about 25 pounds for the average woman.” (1)	Increase focus on weight gain guidelines and effective interventions in medical education.
	Reactive approach	“If they are gaining a lot, then we’ll deal with it.” (2)	Develop practice-level policies that promote appropriate weight gain.
	Lack of accessible resources	“No, there isn’t anything (resources) out there. If you refer to nutrition, they have to pay for it.” (4)	More high quality research on effective interventions for weight management during pregnancy.
Attitudes and Beliefs	Skepticism about counseling’s impact on patient	“I think it is important to talk about why it is healthy for the baby but I think it is good to tell them what your goals are and to make sure you are following it… but I don’t know how much it matters.” (1)	Encourage providers and patients to set appropriate expectations for weight gain. Establish systems to monitor and follow-up so providers and patients can observe results.
	Sensitivity of Topic	“It’s important to try to get a sense of where they are at- their feeling about that (weight gain) because I think you can offend people. Pregnant people are easily offended.” (3)	Educate providers on counseling strategies that are culturally appropriate and sensitive as well as effective.
	Patient more influenced by other factors	“I think there are a lot of cultural issues about expectations and then I think that there is just a common perception you gain as much weight during pregnancy and it doesn’t matter because you’re eating for two.” (8)	Actively involve support people (partners, family, friends) in prenatal care so that others who influence mothers are empowered to ask questions, understand risks, and have the same goals in regards to weight gain.

Each quote is followed by the participant number in parenthesis from whom we quoted.

Several of our findings are consistent with a recent study using focus groups to examine prenatal care providers’ knowledge, attitudes and practices regarding prevention of excessive weight gain during pregnancy [29]. One important difference is that this study sampled providers with expressed interest in weight and nutrition counseling, while our study sampled from a group of providers with no previous knowledge of our research focus. Our findings are similar in that both studies reported a lack of knowledge by providers, a “reactive” approach to excess weight gain, skepticism about counseling’s impact, and perceived sensitivity of the topic. However, one major difference is that our study finds that providers did not prioritize appropriate weight gain highly, while the aforementioned study reports “deep concern” by providers regarding appropriate weight gain [29]. This distinction is likely due to the sampling differences mentioned previously.

Our findings lend support for greater education regarding the morbidity of excess weight gain during pregnancy. In a recent study of obstetric and midwifery staff at a university teaching hospital in Australia, 79% of staff considered their training in advising women about weight gain in pregnancy to be inadequate [30]. For providers, this training should focus on not only the dangers of excess weight gain, but also on ways to approach this topic in a culturally sensitive manner. In addition, greater emphasis on weight gain guidelines during training may benefit providers, while media campaigns focusing on this issue may be effective for patients.

Next, although providers reported that management of weight gain during pregnancy is not a high priority, most providers recognized the importance of diet and exercise and seemed to “try their best” with this approach. What appears to be lacking is effective and affordable interventions to assist both providers and women in managing weight gain during pregnancy as well as research to evaluate the efficacy of those interventions. According to a large prospective cohort study in the US, the advice, when given by providers, has not been shown to influence actual gains during pregnancy [31]. Future studies to further understand barriers to the management of appropriate weight gain can inform more effective interventions. Successful interventions will likely require a team approach including nurses and medical assistants, as often, prenatal education of patients includes these team members. In the meantime, providers must set appropriate expectations and goals for weight gain and follow trends throughout pregnancy. Charts provided to patients at each visit, similar to infant growth charts, might help patients to visualize their weight gain and alert both providers and patients early on about trends towards excess weight gain.

Finally, our findings suggest that providers perceive that patients are influenced more by other factors, such as the patient’s culture, family, and friends. One way to capitalize on this influence is to encourage greater involvement of these support people as shown by studies that recommend that fathers’ needs be assessed and incorporated in a family-oriented approach to prenatal care [32,33]. This would give providers the opportunity to develop meaningful relationships with the patient and those that influence her, and perhaps give more credibility to providers’ advice on weight gain during pregnancy.

Limitations to our study include that a qualitative study is not designed to produce generalizable results beyond the study participants, though as found here, the design does elicit a breadth of opinions about excessive weight gain. Also, our small sample size that included a variety of providers (FP, OB, CNM) with a range of clinical experience from differing sites were analyzed together as part of maximum variation sampling. The location in an academic center where more complex patients tend to accumulate could have contributed to the sense of competing demands with other “more important” or at least “more immediate needs” reported by participants. Finally, we observed that providers often confused excess weight gain during pregnancy with the general concept of obesity during pregnancy and they would often require redirection during interviews.

## Conclusion

Pregnancy represents a critical period when women are at very high risk for transitioning from normal weight to overweight or even obesity. The few months that a woman is pregnant can determine whether she and her child will suffer from the long-term sequelae of obesity and its associated morbidity. Furthermore, pregnancy has been shown to be a time when pregnant women may be especially receptive to behavior change recommendations [34]. This study provides important insight into the challenges prenatal care providers face in managing weight gain during pregnancy. Both providers and patients may benefit from increased awareness of the morbidity of excess weight gain in gestation. Practice-level policies that support the monitoring and management of weight gain during pregnancy could also improve care. These findings should serve as a catalyst for research that further investigates barriers to appropriate weight gain to inform effective interventions.

## Competing interests

The authors declare that they have no competing interests.

## Authors’ contributions

All authors contributed to the conception, design, drafting and revising the manuscript, and have given final approval of the version to be published. TC and ML interviewed the participants, transcribed, coded, and analyzed the qualitative data. All authors read and approved the final manuscript.

## Authors’ information

Tammy Chang, MD, MPH is a Robert Wood Johnson Foundation Clinical Scholar and Clinical Lecturer in the University of Michigan Medical School. She is a family physician who studies obesity in reproductive age women and new media interventions for health promotion.

Mikel Llanes, MD is a Clinical Lecturer in the University of Michigan Medical School. He is a family physician interested in high-risk obstetrical care and preventative healthcare in Latino communities.

Katherine Gold, MD, MSW, MS is an Assistant Professor in the University of Michigan Departments of Family Medicine and the Department of Obstetrics & Gynecology. Her research focuses on pregnancy complications, perinatal loss, and maternal mental health in the peripartum period.

Michael Fetters, MD, MPH, MA serves as Professor of Family Medicine in the University of Michigan. His research interests focus on prevention and the influence of culture on medical decision making, and the application of qualitative and mixed methods research.

## Pre-publication history

The pre-publication history for this paper can be accessed here:

http://www.biomedcentral.com/1471-2393/13/47/prepub
